# High density lipoprotein modulates osteocalcin expression in circulating monocytes: a potential protective mechanism for cardiovascular disease in type 1 diabetes

**DOI:** 10.1186/s12933-017-0599-2

**Published:** 2017-09-16

**Authors:** Ernesto Maddaloni, Yu Xia, Kyoungmin Park, Stephanie D’Eon, Liane J. Tinsley, Ronald St-Louis, Mogher Khamaisi, Qian Li, George L. King, Hillary A. Keenan

**Affiliations:** 1 Research Division, Joslin Diabetes Center, Harvard Medical School, One Joslin Place, Boston, MA 02215 USA; 2 Department of Medicine, Unit of Endocrinology and Diabetes, University Campus Bio-Medico, Rome, Italy

**Keywords:** Osteocalcin, Type 1 diabetes, Cardiovascular disease, HDL, Monocytes, Calcifying monocytes

## Abstract

**Background:**

Cardiovascular disease (CVD) is a major cause of mortality in type 1 diabetes (T1D). A pro-calcific drift of circulating monocytes has been linked to vascular calcification and is marked by the surface expression of osteocalcin (OCN). We studied OCN+ monocytes in a unique population with ≥50 years of T1D, the 50-Year Joslin Medalists (J50M).

**Methods:**

CD45 bright/CD14+/OCN+ cells in the circulating mononuclear blood cell fraction were quantified by flow cytometry and reported as percentage of CD45 bright cells. Mechanisms were studied by inducing OCN expression in human monocytes in vitro.

**Results:**

Subjects without history of CVD (n = 16) showed lower levels of OCN+ monocytes than subjects with CVD (n = 14) (13.1 ± 8.4% vs 19.9 ± 6.4%, p = 0.02). OCN+ monocytes level was inversely related to total high density lipoprotein (HDL) cholesterol levels (r = −0.424, p = 0.02), large (r = −0.413, p = 0.02) and intermediate (r = −0.445, p = 0.01) HDL sub-fractions, but not to small HDL. In vitro, incubation with OxLDL significantly increased the number of OCN+ monocytes (p < 0.01). This action of OxLDL was significantly reduced by the addition of HDL in a concentration dependent manner (p < 0.001). Inhibition of the scavenger receptor B1 reduced the effects of both OxLDL and HDL (p < 0.05).

**Conclusions:**

Low OCN+ monocytes levels are associated with lack of CVD in people with long duration T1D. A possible mechanism for the increased OCN+ monocytes could be the elevated levels of oxidized lipids due to diabetes which may be inhibited by HDL. These findings suggest that circulating OCN+ monocytes could be a marker for vascular disease in diabetic patients and possibly modified by HDL elevation.

**Electronic supplementary material:**

The online version of this article (doi:10.1186/s12933-017-0599-2) contains supplementary material, which is available to authorized users.

## Background

Type 1 diabetes (T1D) is a disease characterized by hyperglycemia due to autoimmune destruction of pancreatic beta-cells. Several end-organ complications develop as a consequence of chronic exposure to hyperglycemia. Among these, cardiovascular disease (CVD) is the major cause of decreased life expectancy [1]. Factors other than hyperglycemia also contribute to the pathogenesis of CVD in T1D. In particular inflammation and dyslipidemia play a pivotal role in the development of atherosclerotic diseases [2–4]. Overall, an imbalance between mechanisms of injury and protective factors contributes to vascular complications in diabetes [5]. In particular, our recent data suggest circulating progenitor cells are associated with protection from CVD in subjects with long-standing T1D [6]. On the other hand, circulating osteoprogenitor cells, defined as circulating cells co-expressing osteocalcin (OCN) together with the progenitor stem cell antigen CD34, have been found increased in subjects with cardiovascular disease with and without diabetes [7]. Because of their pro-calcific phenotype, these cells are hypothesized to contribute to the development of vascular calcification and atherosclerosis [8]. Recently, the differentiation towards a pro-calcific phenotype of circulating monocytes has been related to vascular calcification and CVD in those with type 2 diabetes (T2D) [9, 10]. The surface expression of the bone-related protein OCN is the first and essential marker of this drift [9, 11]. Growing evidence demonstrates the ability of circulating OCN+ mononuclear cells to contribute to ectopic ossification [12–14].

Some clinical and pathological features of CVD differ between T1D and T2D [15]. Following this, OCN+ monocytes have not yet been explored in T1D. In addition, characterizing factors modulating the expression of OCN in monocytes, especially in diabetes could be important. Oxidized low density lipoprotein (OxLDL) is a known activator of monocytes which are involved in the pathogenesis of atherosclerosis. OxLDL can also induce osteogenic differentiation of different cell types including smooth muscle and endothelial cells [16, 17]. As monocytes are the primary target cells of oxidized lipids, we hypothesized that OxLDL could induce OCN expression in these cells. Moreover, as high density lipoprotein (HDL) actively interacts with monocytes to protect from the development of CVD we hypothesized HDL may counteract the effects of oxidized lipids. We previously identified a cohort of subjects, who were protected from clinically significant CVD after 50 years or more of T1D (50-Year Medalists) which appeared to be associated with elevated HDL-c levels [18]. Therefore the hypothesis of whether OCN+ monocytes were related to CVD and HDL-c was tested in this cohort, then the action of OxLDL and HDL on OCN expression and possible mechanisms were characterized in vitro.

## Methods

### Study population

Details of the 50-Year Medalist Study and its methods have been extensively described elsewhere [19–22]. In brief, participants have 50 or more years of documented insulin dependence since time of diagnosis. All individuals were assessed at the Joslin Diabetes Center in Boston, MA, by clinical exam, electrocardiogram, and standard laboratory measures. Thirty-three consecutive Medalist Study participants were enrolled from March 2015 to November 2015 and screened for this sub-study. Subjects with chronic immobilization, hematologic or neoplastic diseases in progress, history of hyper- or hypoparathyroidism were excluded (n = 3) from the study. Positive cardiovascular history was defined as self-reported history of coronary angioplasty, cardiac bypass surgery, hospitalization for heart attack, leg bypass surgery, leg angioplasty or stroke [21]. Other diabetic complications were assessed according to pre-specified criteria [23, 24] (Additional file 1: Additional methods). Urine and blood specimens were collected for biochemical assays (Additional file 1: Additional methods). The Lipoprint system (Quantimetrix, Inc., Redondo Beach, CA) was used to assess HDL and LDL subfractions distinguishing ten HDL and seven LDL subfractions. HDL subfractions were grouped into 3 categories: large (HDL 1–3), intermediate (HDL 4–7), and small (HDL 8–10) [25]. Small and dense LDL were identified as LDL 3-7 fractions [26].

### Identification and quantification of circulating OCN+ monocytes by flow cytometry

Based on previous reports [10, 27], OCN+ monocytes were searched in the peripheral blood mononuclear cells (PBMCs) fraction. Blood samples were collected while subjects were fasting and peripheral blood mononuclear cells (PBMCs) were isolated within 2 h (Additional file 1: Additional methods). Freshly isolated PBMCs were washed 3 times in phosphate-buffered saline (PBS) with 1% fetal bovine serum (FBS) and then incubated for 45 min at 4 °C in the dark with BrilliantViolet421-conjugated anti-human CD45 (BioLegend, San Diego, CA), PE/Dazzle594-conjugated anti-human CD14 (BioLegend, San Diego, CA) and AlexFluor488-conjugated anti-human osteocalcin antibodies (R&D Systems, Minneapolis, MN), according to the manufacturers’ instructions. All antibodies were titrated to achieve working concentrations. After incubation samples were washed other three times in PBS with 1% FBS and then assessed by flow cytometry. Ten minutes before cell counts, cells were stained for viability with 7-aminoactinomycin D (7AAD). Up to one million events were recorded for each sample. Data were analyzed with FlowJo software (Tree Star, Ashland, OR) according to the gating strategy described in Additional file 1. Samples were processed in duplicate; the mean of two runs was used as levels of circulating OCN+ monocytes.

### THP-1 and U937 culture and treatments

THP-1 and U937 cells (ATCC^®^ TIB-202 and ATCC^®^ CRL-1593.2 Manassas, VA) were treated with 40 μg/ml Oxidized-LDL (OxLDL) ± HDL ± 40 μg/ml LDL (AlfaAesar, ThermoFisher Scientific, Waltham, MA). The chemical inhibitor BLT-1 (SML0059, Sigma, St. Louis, MO) and scavenger receptor, class B, type I (SR-BI) specific blocking antibody (NB400-101, Novus Biologicals, Littleton, CO) were used to inhibit the SR-B1. Anti-rabbit IgG (Sigma, St. Louis, MO) was used as a control Ab. Cells were treated with 0.25 μM BLT-1 or SR-BI antibody (1:800 and 1:500, respectively) for 1 h, and then OxLDL and/or HDL were added as described above.

### Assessment of osteocalcin expression in THP-1 and U937 cells

Osteocalcin expression in THP-1 and U937 cells was assessed by immunoblot analysis and by flow cytometry as described in Additional file 1: Additional methods. OCN mRNA was assessed by quantitative real time polymerase chain reaction (qRT-PCR) as described in Additional file 1: Additional methods.

### Assessment of nuclear levels of run-related transcription factor 2 in THP-1 and U937 cells

Nuclear and cytosolic fractions were isolated from THP-1 and U937 cells using the Compartment Protein Extraction Kit (Millipore, Cat#2145, Billerica, MA). Nuclear and cytosolic protein concentrations were measured using the Bradford assay. The proteins were blotted with an antibody specific for RUNX2 and Lamin B1 purchase from Abcam (Cambridge, MA) at 1:1000 dilution.

### Statistical analysis

Values are expressed as mean ± SD or as medians (25th–75th percentile range) for continuous variables and as proportions for categorical variables (%). Variables were tested for normality using the Shapiro–Wilk test. Comparisons were done using Student’s t test, Kruskal–Wallis, and Chi square or Fisher exact test depending on distribution and sample size. One-way, two-way or three-way analysis of variance (ANOVA) were used as appropriate. Correlations were tested by Pearson or Spearman test depending on distribution. Linear models were used for multivariable analyses to adjust for covariates, with p < 0.05 considered significant for testing in the final model with main effect and outcome. All statistical analyses were performed using Stata/IC 12.1 software (StataCorp, College Station, TX).

## Results

### Population features

Thirty-three consecutive subjects enrolled in the 50-Year Joslin Medalist Study were screened for participation in this study and three excluded according to the pre-specified exclusion criteria: two for hematologic diseases (chronic lymphocytic leukemia and lymphoma), and 1 due to hyperparathyroidism, leaving 14 males and 16 females eligible. Population features in the whole population and by CVD are summarized in Table 1. For complications, 14 (46.7%) had reported CVD, 18 (60.0%) had diabetic retinopathy, three (10.0%) had nephropathy and 15 (50.0%) had neuropathy. Twenty-three (76.7%) subjects were on lipid lowering agents, 20 (66.7%) on anti-hypertensive medications and 3 (10.0%) on anti-osteoporotic drugs. There were no differences in gender, age, disease duration and anthropometric parameters between subjects with and without CVD. Median [Q1–Q3] HbA1c was 6.9% [6.6–7.3], and similar between subjects with and without CVD (p = 0.92). Those with and without CVD did not have significant differences in eGFR (69.5 [53.0–90.4] vs 92.0 [72.0–96.4] ml/min/1.73 m^2^, p = 0.093). HDL cholesterol levels trended higher (65 [55–85] vs 61 [45–71] mg/dl, p = 0.09) while intermediate HDL sub-fractions were significantly higher in those without CVD (29.6 ± 7.1 vs 24.3 ± 4.6 mg/dl, p = 0.03). Yet, no significant differences were found in large (32.1 ± 12.5 vs 27.0 ± 11.5 mg/dl, p = 0.26) and small HDL sub-particles (8.6 ± 2.6 vs 7.6 ± 1.7 mg/dl, p = 0.20). Total cholesterol, triglycerides, LDL, 25-OH vitamin D, calcium, alkaline phosphatase and hs-CRP levels did not differ between those with and without CVD. Additionally, no differences in these markers were found by sex. A higher level of large particle HDL was found in females relative to males (34.1 ± 11.7 vs 24.8 ± 11.0 mg/dl, p = 0.03). Yet, no differences by sex were found in intermediate (28.5 ± 6.6 vs 25.5 ± 6.3 mg/dl, p = 0.22) and small HDL (7.8 ± 2.5 vs 8.6 ± 1.9 mg/dl, p = 0.32).

**Table 1:** Population features

	Overall n = 30	No CVD n = 16	CVD n = 14	p value between CVD status
Gender, males/females	14/16	6/10	8/6	0.210
Age, years	64 [59–71]	62 [59–68]	68 [64–73]	0.257
Disease duration, years	56 [51–62]	55 [51–60]	56 [52–64]	0.333
BMI, kg/m^2^	26.1 [22.9–28.9]	26.3 [22.4–28.1]	26.1 [23.9–30.1]	0.863
Waist to hip ratio	0.9 [0.8–0.9]	0.9 [0.8–0.9]	0.9 [0.8–0.9]	1.000
Insulin dose, IU/kg	0.45 [0.38–0.58]	0.46 [0.41–0.58]	0.44 [0.36–0.54]	0.830
eGFR, ml/min/1.73 m^2^	86.8 [60.0–95.6]	92.0 [72.0–96.4]	69.5 [53.0–90.4]	0.093
HbA1c, %	6.9 [6.6–7.3]	6.9 [6.3–7.4]	6.9 [6.6–7.2]	0.922
Total cholesterol, mg/dl	152 [140–167]	160 [140–176]	146 [123–157]	0.275
Triglycerides, mg/dl	63 [46–80]	60 [45–75]	65 [50–86]	0.236
HDL-c, mg/dl	64 [52–76]	65 [55–85]	61 [45–71]	0.091
LDL-c, mg/dl	68 [61–86]	69 [63–90]	67 [60–85]	0.901
Triglycerides/HDL ratio	1.00 [0.64–1.34]	0.80 [0.56–1.33]	1.15 [0.72–1.43]	0.155
Corrected calcium, mg/dl	9.0 [8.8–9.2]	9.0 [8.8–9.3]	9.0 [8.8–9.1]	0.956
25-OH vitamin D, ng/ml	40 [31–44]	40 [29–46]	40 [32–44]	0.906
Alkaline phosphatase, U/l	62 [46–81]	59 [52–83]	66 [45–78]	0.921
hsCRP, mg/dl	0.14 [0.10–0.26]	0.13 [0.10–0.37]	0.15 [0.10–0.24]	0.630

Values are median [Q1–Q3] for continuous variables and numbers for categorical variables

*CVD* cardiovascular disease, *BMI* body mass index, *eGFR* estimated glomerular filtration rate, *HDL-c* high density lipoprotein cholesterol, *LDL-c* low density lipoprotein cholesterol, *hsCRP* high sensitivity C-reactive protein

### OCN+ monocytes levels differ by CVD and its risk factors

**Fig. 1:**
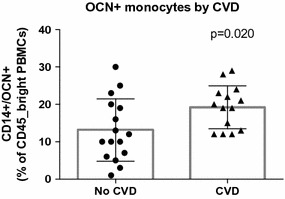
OCN+ monocytes by presence of cardiovascular disease. OCN+ monocytes are expressed as percentage of CD45_bright PBMCs. Subjects without history of CVD showed lower levels of circulating MCC

Subjects without CVD showed significantly lower levels of circulating CD45_bright/CD14+/OCN+ cells than subjects with CVD (13.1 ± 8.4% vs 19.9 ± 6.4%, p = 0.02) (Fig. 1). No significant differences were found in the overall levels of CD45_bright and CD45_bright/CD14+ cells between CVD groups indicating no bias in the overall number of cells (Additional file 2: Figure S1a, b). Three subjects with overt diabetic nephropathy had reported history of CVD and corresponding higher levels of CD45_bright/CD14+/OCN+ cells compared to subjects without nephropathy (27.3 ± 3.1% vs 15.1 ± 7.6%, p = 0.03) (Additional file 3: Figure S2A). Additionally, circulating levels of CD45_bright/CD14+/OCN+ were neither associated with proliferative diabetic retinopathy (p = 0.31) nor neuropathy (p = 0.53) (Additional file 3: Figure S2B, C).

**Fig. 2:**
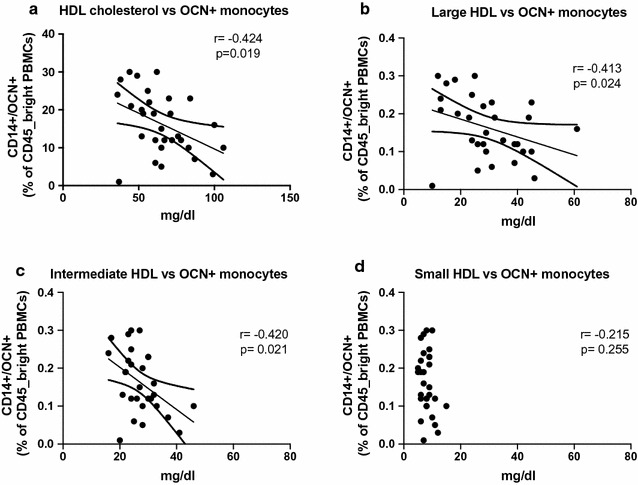
Correlation between HDL cholesterol and HDL sub-fractions with OCN+ monocytes. OCN+ monocytes are expressed as percentage of CD45_bright PBMCs. Total, large and intermediate, but not small HDL were inversely related to OCN+ monocytes

As the clinical relationship of HDL and CVD was further explored with levels of circulating CD45_bright/CD14+/OCN+, we found these cells were significantly and inversely associated to total HDL cholesterol levels (r = −0.424, p = 0.019) (Fig. 2a). Additionally, similarly to the analysis of CVD, examination of HDL sub-fractions showed the levels of OCN+ cells were inversely related to the favorable large (r = −0.413, p = 0.02) and intermediate (r = −0.445, p = 0.01) subfractions, while no significant relationship was found with small subfraction levels (Fig. 2b–d). Differently from HDL, CD45_bright/CD14+/OCN+ cell levels were not related to total cholesterol, LDL cholesterol and triglycerides. However, the analysis of LDL subfractions showed a trend towards a positive association between small and dense LDL and CD45_bright/CD14+/OCN+ cell levels (r = 0.336, p = 0.07).

In parallel with the above, CD45_bright/CD14+/OCN+ cell levels were not related to age, disease duration, glycemic control, renal function, calcium, 25-OH vitamin D, alkaline phosphatase or hs-CRP (Additional file 4: Table S1). The use of lipid lowering agents and anti-hypertensive drugs was also not associated with the levels of CD45_bright/CD14+/OCN+ cells.

### OxLDL and HDL action on osteocalcin expression in monocyte cell lines through SR-B1

**Fig. 3:**
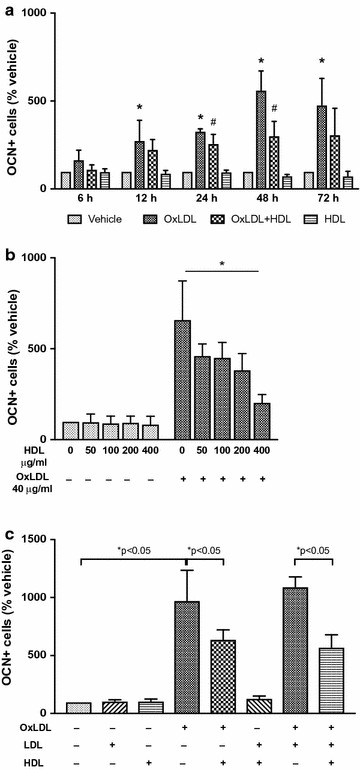
Effect of OxLDL, LDL and HDL on OCN surface expression in THP-1 cells. **a** OCN + THP1 cells were measured by flow cytometry after treatment with 40 μg/ml OxLDL ± 200 μg/ml HDL at different time points (n = 4 in each group). Three-way ANOVA for multiple comparisons: p < 0.001. Kruskal–Wallis test for pairwise comparison: *p < 0.05 vs untreated; ^#^p < 0.05 vs OxLDL treated. **b** OCN + THP1 cells were measured by flow cytometry after treatment with 40 μg/ml OxLDL and different concentrations of HDL for 48 h (n = 5 in each group). Two-way ANOVA for multiple comparisons: *p < 0.001. **c** OCN + THP1 cells were measured by flow cytometry after 48 h treatment with 40 μg/ml OxLDL ± 200 μg/ml HDL ± 40μg/ml LDL (n = 3 in each group). Three-way ANOVA for multiple comparison: p < 0.001. *p values at the Kruskal–Wallis test for pairwise comparisons

To evaluate a possible direct interaction between HDL and expression of OCN, we studied its expression by HDL and OxLDL in THP-1 cells, a human monocyte cell line. After treatment with 40 μg/ml OxLDL for 12, 24, 48 and 72 h, the number of THP-1 cells expressing osteocalcin, as evaluated by flow cytometry, significantly increased three to tenfolds (p < 0.001) (Fig. 3a–c).

To determine whether HDL can directly modify the expression of OCN, we incubated THP1 cells with HDL at a starting concentration of 50 μg/ml and titrated up to 400 μg/ml with and without OxLDL (Fig. 3b). HDL alone had no effects on the number of OCN+/THP1 cells. A 30% reduction in OxLDL stimulated-OCN+/THP1 cells after 48 h exposure to 50 and 100 μg/ml HDL, 40% reduction with 200 μg/ml HDL and approximately 70% reduction with 400 μg/ml (p < 0.001) were observed (Fig. 3b). Time course and dose-dependent studies showed the effect increased with incubation time and dose of HDL (Fig. 3a, b). Henceforth, stimulation experiments were performed by incubating cells for 48 h. with 200 μg/ml of HDL, a condition which mimicked human physiology [28].

To evaluate whether non-oxidized LDL may affect OCN expression, THP1 cells were incubated for 48 h with 40 μg/ml LDL with and without OxLDL and HDL (Fig. 3c). Differently from OxLDL, non-oxidized LDL did not change the expression of OCN and did not interact with HDL.

**Fig. 4:**
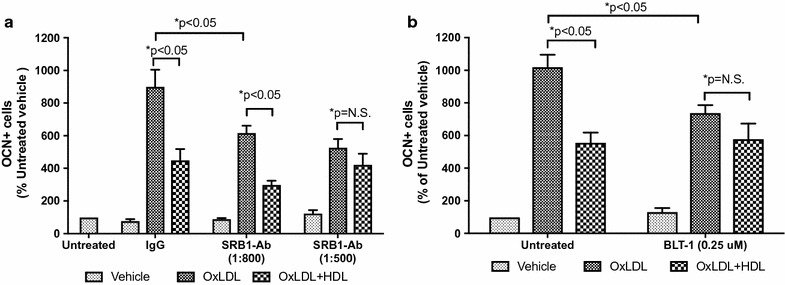
Inhibition of the scavenger receptor B1. Treatment of THP-1 cells with inhibitors of SR-B1–SRB1-Ab (**a**) and BLT1 (**b**)—mitigates the effects of both OxLDL and of HDL in terms of OCN+ cell as evaluated by flow cytometry. Three-way ANOVA: p < 0.001. *p values at the Kruskal–Wallis test for pairwise comparisons

To better characterize the effect of HDL on OCN+ cells, the effect of SR-B1 inhibition was studied. As shown in Fig. 4, the number of OCN+ cells was increased by incubation with OxLDL by 9 (±2) fold and were reduced by 50.1% (±9.3%) in the presence of HDL. The addition of SR-B1 Ab at 1:800 and 1:500 decreased the effect of OxLDL by 29.8% (±9.9%) and 39.2% (±15.7%), which was similar to HDL above. Similar results were obtained using BLT-1, a small molecule inhibitor of HDL’s actions via HDL receptor SR-B1 (Fig. 4).

**Fig. 5:**
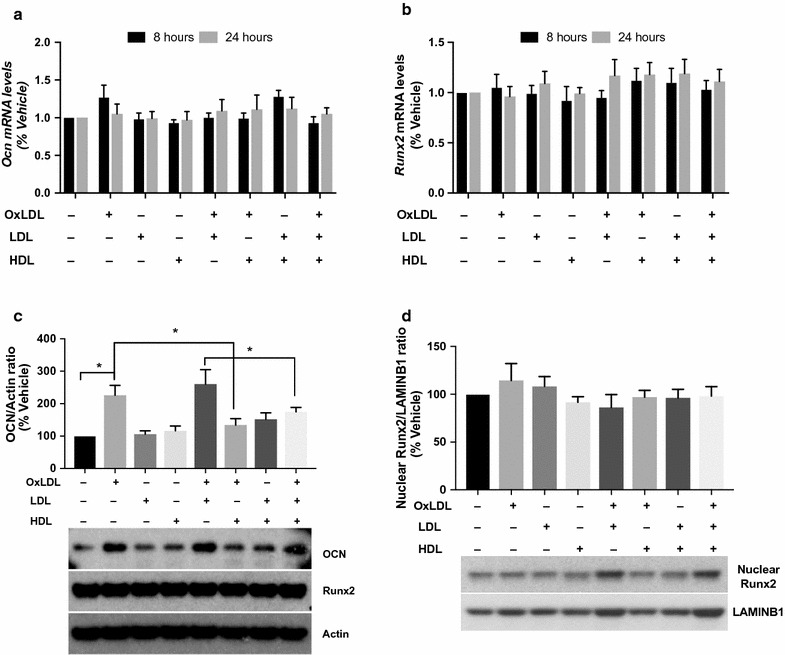
Effect of OxLDL, LDL and HDL on OCN and Runx2 gene and protein expression in THP1 cells. mRNA and protein levels were quantified after treatment with 40 μg/ml OxLDL ± 200 μg/ml HDL ± 40μg/ml LDL for 8 and 24 h (RT-PCR) or for 48 h (immunoblot). OCN (**a**) and Runx2 (**b**) mRNA levels (n = 3 for each group) did not change after 8 or 24 h incubation with OxLDL, LDL or HDL. Three-way ANOVA for multiple comparison: p > 0.05. **c** A representative immunoblot is shown for both OCN and Runx2 proteins. The bar graph shows the quantification of the immunoblot for OCN (n = 3 in each group). OCN protein level significantly increased after incubation with OxLDL alone, but not after incubation with OxLDL + HDL. LDL did not affect OCN levels. Three-way ANOVA for multiple comparison: p < 0.001. *p < 0.05 at the Kruskal–Wallis test for pairwise comparisons. Quantification of Runx2 expression did not show significant changes between groups (data not shown). **d** Nuclear Runx2 protein levels did not change after incubation with OxLDL, HDL or LDL (n = 3 in each group). Three-way ANOVA for multiple comparison: p > 0.05

The increases in OCN expression on circulating monocytes induced by OxLDL appears to be at the protein level since *Ocn* mRNA levels were not changed by exposure to OxLDL, LDL or HDL (Fig. 5a). However, total protein levels of OCN in monocytes were significantly increased by OxLDL and returned to baseline by the addition of HDL, while LDL alone did not affect OCN protein in monocytes (Fig. 5c). Consistent with the absence of changes in gene expression, no significant differences in the expression of the Run-related transcription factor 2 (Runx2), a major regulator of the OCN gene, was observed both at the gene and protein levels (Fig. 5b–d).

Experiments were repeated using another monocyte cell line (U937). The results of the new experiments confirming those from the previous work are reported in Additional file 5: Figure S3 and Additional file 6: Figure S4.

## Discussion

In this study a reduced number of OCN+ monocytes was associated with reduced prevalence of cardiovascular disease and higher HDL-c levels. In addition, data from cellular models support the clinical finding HDL significantly reduces the number of monocytes expressing OCN due to OxLDL by interacting with SR-B1, a receptor already known for interacting with HDL to effect cholesterol efflux [29]. Overall, this suggests a potential link between increased HDL-c and lowering the risk of cardiovascular disease by decreasing the expression of OCN in monocytes.

Eghbali-Fatourechi et al. reported circulating osteoblast progenitors as mononuclear circulating OCN+ cells are able to form mineralized nodules when cultured in osteoblast-differentiating medium [13]. Subsequently other investigators have confirmed that circulating mesenchymal osteoprogenitors ability to cause ectopic vascular calcification [30–33]. Monocytes have recently been described as a source of mesenchymal progenitors which can differentiate into osteoblast-like cells [8, 34] contributing to atherosclerotic calcification [14], and some reports have suggested circulating myeloid cells with osteogenic potential may affect CVD in the general population [9, 10]. Our studies extend the association of OCN+ monocytes and CVD in T1D which while having similarities with T2D also differs in that individuals with T1D develop CVD often without the typical insulin resistance hallmarks seen in T2D [15].

OCN is the most abundant non-collagenous bone matrix protein, with several functions beyond skeletal health. In particular, circulating levels of serum osteocalcin have been associated with both glucose metabolism [35–37] and cardiovascular disease [38, 39], but with contrasting results. As a bone-related protein, OCN is mainly produced and secreted by osteoblasts. However, different cell types involved in CVD express OCN, such as platelets, monocytes and endothelial progenitor cells [8, 40]. The presence in the bloodstream of circulating cells with an osteogenic phenotype linked to CVD supports the existence of a bone-vascular axis we have recently described also in the Medalists [22]. Therefore, further studies should be designed to clarify whether an association exists between markers of bone health (bone-turnover markers, bone mineral density, etc.) and circulating osteogenic cells.

Here we propose a mechanism which stimulates the pathological expression of OCN in circulating monocytes and a means to reverse it, with support from in vitro experiments. Accumulated OxLDL in the arterial walls may lead to vascular calcification by inducing the trans-differentiation of vascular smooth muscle cells into calcifying cells through the upregulation of Runx2, which is a major regulator of osteoblast differentiation [16, 41]. Our data show OxLDL promotes the pro-calcific phenotypic drift of monocytes as well, demonstrated by an increased number of cells expressing OCN on the cell surface. However, this does not seem related to genetic regulation as neither changes in *Ocn* mRNA levels nor in the expression of Runx2, the main transcription factor regulating *Ocn* were seen; thus suggesting other mechanisms causing a change in OCN protein levels and surface expression not accompanied by gene expression change. One possibility is protein recycling which has been associated with HDL action on monocytes [29, 42]. Alternatively, the changes observed in protein levels by immunoblot could also be due to post-translational processing of OCN regulated by HDL and OxLDL. Further studies should be performed to clarify this issue.

Consistently with the absence of relationships between LDL cholesterol levels and CD45_bright/CD14+/OCN+ cell levels in the Medalists, in vitro studies also showed non-oxidized LDL have no effect on OCN expression. However, we describe a trend towards a positive association between CD45_bright/CD14+/OCN+ circulating cell levels and small and dense LDL, but not with large LDL. This is consistent with the in vitro study as small and dense LDL represent the LDL fractions more prone to oxidation with higher atherogenic properties than LDL particles with higher size and lower density [43, 44].

Other mechanisms we did not investigate here may lead to the induction of osteogenic drift of monocytes and should be searched in future studies. Overall, it is evident that different stimuli such as hyperglycemia, dyslipidemia, chronic inflammation and hypoxia [7–9], all of which are pathologically elevated in people affected by diabetes, combine leading to the pro-calcific milieu contributing to the high prevalence of vascular calcification observed in people with diabetes.

Importantly, our data show both in vivo and in vitro, HDL is a relevant factor which can counteract the increased expression of OCN. The inverse relationship between OCN+ monocytes and HDL-c suggests a potential new mechanism for HDL to lower the risk for CVD. In particular the larger and intermediate sub-particles of HDL, which facilitate cholesterol efflux and are associated with lower cardiovascular risk [45–47], are correlated with lower levels of OCN+ cells. This supports our previous epidemiologic work demonstrating protection from CVD in the 50-Year Medalist is associated with HDL-c levels [18]. This is consistent with several other studies reporting an inverse relationship between HDL cholesterol levels and CVD in those with type 2 diabetes and those with the disease [48, 49]. However, interventional trials aimed at increasing HDL-c levels have not shown success in decreasing cardiovascular events [50], highlighting the importance of a greater mechanistic understanding of HDL-mediated cardiovascular protection. The interaction between HDL and monocytes/macrophages has always been considered a key for the maintenance of healthy vessels. Previously, this relationship was confined to HDL’s effect on cholesterol efflux and anti-inflammatory actions, preventing foam cell formation and inflammation [51]. Based on our results, we propose a new mechanism by which HDL and monocytes interact to protect from cardiovascular disease by counteracting monocytes differentiation into pro-calcific cells.

The surface receptors ATP-binding cassette A1 and C1 and SR-B1 are the main molecules mediating the action of HDL on monocytes/macrophages. Of these, SR-B1 appears to contribute both to the mechanisms of cholesterol diffusion and retro-endocytosis [29]. Our data suggests SR-B1 is also a key receptor in the regulation of OCN expression by both OxLDL and HDL, providing a potential target to reduce vascular stiffness and/or increase plaque stability.

A bias of the study may be that it was done in an older population with T1D who have a median age of 65 years. However, this is an advantage as it is a population who have reached their end-phenotype- those who will or will not develop significant CVD. The extreme phenotype provides more power with a smaller sample size. This provides added value as current knowledge about CVD in T1D is from studies conducted in the previous era of less intensive glycemic control, in those of lesser duration or extrapolated from studies in T2D [15]. Moreover, the changing epidemiology of T1D, characterized by increased longevity and longer disease duration without complications increases the need for understanding of what makes survival possible, particularly for CVD, the largest cause of mortality among this group [52]. Yet, these findings were confirmed in via in vitro experiments performed on two different types of monocyte cell lines (THP-1 and U937) and not on primary monocytes from this selected population. THP-1 and U937 cells have been widely used to investigate monocytes/macrophages pathophysiology in the cardiovascular system and it has been shown that these cell lines have features of primary monocytes derived from control human donors [53]. The absence of a control group of healthy subjects without T1D may also limit our study. However, previous studies have already widely shown that non-diabetic people have lower levels of OCN+ monocytes [7, 9, 10]. While the main mechanism of vascular damage associated to OCN+ monocytes should be ectopic calcification in the vessel wall, we acknowledge that the lack of direct measurements of vascular calcification in the Medalist cohort should be considered as a limit of this study. However, this was not the primary aim of our study as previous studies already investigated the contribution of OCN+ monocytes to vascular calcification [13, 14] and it has been widely demonstrated that vascular calcification is among the main pathological findings in T1D with CVD [15].

## Conclusions

In conclusion, this study supports an association between CVD protection and lower levels of circulating osteogenic cells of myeloid origin in long duration T1D, along with higher HDL-c levels, particularly those of larger sub-particle size. Our data suggest a mechanism for the increased OCN+ monocytes due to oxidized lipids found in diabetes, and that this may be mitigated by HDL. These findings indicate that circulating OCN+ monocytes may be a marker for vascular disease in diabetic patients and may be modified by HDL elevation. Results regarding the regulation of OCN expression on monocytes by OxLDL and HDL through SR-B1 and its relationship with CVD in T1D provide new information on vascular pathophysiology. Indeed, these findings may provide new insights on the mechanism of HDL-mediated cardiovascular protection and promote advances in therapeutic strategies.

## Additional files

**Additional file 1.** Additional methods.

**Additional file 2: Figure S1.** CD45_bright and CD45_bright/CD14+ PBMCs by presence of cardiovascular disease. No differences were found between Medalists with and without history of cardiovascular disease.

**Additional file 3: Figure S2.** OCN+ monocytes by complications other than cardiovascular disease.

**Additional file 4: Table S1.** Linear regression analysis for association between CD45_bright/CD14+/OCN+ (dependent variable) and different clinical and biochemical factors (independent variables).

**Additional file 5: Figure S3.** OCN surface expression in U937. A. OCN+ U937 cells were measured by flow cytometry after 48 h treatment with 40 μg/ml OxLDL ± 200 μg/ml HDL ± 40μg/ml LDL (n = 3 in each group). Three-way ANOVA for multiple comparison: p < 0.001. *p < 0.05 at the Kruskal–Wallis test for pairwise comparisons. B and C. Treatment of U937 cells with inhibitors of SR-B1–SRB1-Ab (B) and BLT1 (C)—mitigates the effects of both OxLDL and of HDL in terms of OCN+ cell as evaluated by flow cytometry. Three-way ANOVA: p < 0.001. *p values at the Kruskal–Wallis test for pairwise comparisons.

**Additional file 6: Figure S4.** OCN and Runx2 gene and protein expression in U937 cells. A, B. OCN and Runx2 mRNA levels (n = 3 for each group). Three-way ANOVA for multiple comparison: p > 0.05. C. A representative immunoblot is shown for both OCN and Runx2 proteins. The bar graph shows the quantification of the immunoblot for OCN (n = 3 in each group). OCN protein level significantly increases after incubation with OxLDL alone, but not after incubation with OxLDL + HDL. Three-way ANOVA for multiple comparison: p < 0.001. *p < 0.05 at the Kruskal–Wallis test for pairwise comparisons. Quantification of Runx2 expression did not show significant changes between groups (data not shown). D. Nuclear Runx2 protein levels does not change after incubation with OxLDL, HDL or LDL (n = 3 in each group). Three-way ANOVA for multiple comparison: p > 0.05.

## Abbreviations

T1D: type 1 diabetes
CVD: cardiovascular disease
OCN: osteocalcin
T2D: type 2 diabetes
OxLDL: oxidized low density lipoprotein
HDL: high density lipoprotein
HDL-c: high density lipoprotein cholesterol
PBMCs: peripheral blood mononuclear cells
PBS: phosphate-buffered saline
FBS: fetal bovine serum
7AAD: 7-aminoactinomycin D
SR-BI: scavenger receptor, class B, type I
qRT-PCR: quantitative real time polymerase chain reaction
ANOVA: analysis of variance
