# Supplementary material for: High density lipoprotein modulates osteocalcin expression in circulating monocytes: a potential protective mechanism for cardiovascular disease in type 1 diabetes
Source: Cardiovasc Diabetol. 2017 Sep 16;16:116. doi: 10.1186/s12933-017-0599-2 (PMC5602856; doi:10.1186/s12933-017-0599-2)
Supplement: Supplementary file 1 — Additional file 1. Additional methods. [file 12933_2017_599_MOESM1_ESM.docx]

**Additional Methods**

**Assessment of diabetes complications**

Diabetic complications were assessed according as follows: (I) positive cardiovascular history was defined as self-reported history of coronary angioplasty, cardiac bypass surgery, hospitalization for heart attack, leg bypass surgery, leg angioplasty or stroke [1]; (II) peripheral neuropathy was assessed using the Michigan Neuropathy Screening Index (MNSI; score≥2) [2]; (III) nephropathy was defined as an albumin-to-creatinine ratio (ACR) >30 mg/g and an estimated glomerular filtration rate (eGFR) <60 mL/min per 1.73 m^2^ [3]; (IV) diabetic retinopathy was diagnosed using a seven-standard field fundus photography and graded according the Early Treatment Diabetic Retinopathy Study (ETDRS >53) [4]

**Biochemical assays**

HbA1c was assessed by Immunoturbidimetry; high-sensitivity C-reactive protein (CRP) was determined by nephelometric methods; creatinine, calcium, albumin, total cholesterol, high density lipoprotein (HDL) cholesterol, triglycerides were assessed by spectrophotometry and LDL cholesterol calculated; urine ACR by turbidimetric method (Quest Diagnostics, Wallingford, CT).

**PBMCs isolation**

Fasting blood samples were collected into cell preparation tubes with sodium citrate for the separation of mononuclear cells from whole blood (BD Vacutainer^®^ CPT™). After collection, tubes were stored upright at room temperature until centrifugation. Blood samples were centrifuged within two hours from collection at room temperature (18-25ºC) in a horizontal rotor (swing-out head) for a minimum of 20 minutes at 2800 rpm (low brake). Immediately following centrifugation the mononuclear cell layer (buffy coat) was collected with a Pasteur pipette and transferred to a 15 mL size conical centrifuge tube for washing steps. Cell washing: cells were resuspended to 10mL with PBS+1%FBS and then centrifuged for 8 minutes at 1500rpm at 10°C with full bracke and acceleration.

**Gating strategy for the identification and quantification of circulating OCN+ monocytes by flow-cytometry.**

Data were analyzed with FlowJo software (Tree Star, Ashland, OR) according to the following gating strategy. Doublets were excluded by a FSC-W vs FSC-A scatter. A side and forward scatter was used for a first identification of lymphocytes and monocytes by size and granularity. After exclusion of nonviable cells by gating cells negative for 7AAD, we gated CD45 bright cells and then examined the expression of CD14 and osteocalcin. Fluorescence minus one (FMO) controls were used to optimize the gating strategy. OCN+ monocytes were quantified as CD45_bright PBMCs positive for CD14 and OCN and are expressed as percentage of CD45_bright.

**Media used for cell cultures**

THP-1 cells (ATCC^®^ TIB-202, Manassas, VA, USA) were cultured in low glucose RPMI-1640 containing 10% heat-inactivated FBS, 100 IU/ml penicillin, and 100 μg/ml streptomycin.

**Immuno-blot analysis**

Cells were harvested and lysed in RIPA lysis buffer. The protein concentration was quantified using the bicinchoninic acid (BCA) assay kit (Thermo Fisher Scientific). Equivalent proteins were loaded onto 4-20% gradient native gel (criterion, Bio-Rad) and subsequently electrotransferred onto nitrocellulose membranes. The membranes were blocked with 5 % skim milk and incubated overnight at 4℃ with anti-Osteocalcin antibody (1:1000; AB10911, EMD Millipore). After washing with TBS-T, the membranes incubated with secondary antibody (1:3000, Cell Signaling) for 1 h at room temperature. Finally, the membranes were visualized using enhanced chemiluminescence (ECL) kit (Thermo Fisher Scientific) and quantified using ImageJ.

**qRT-PCR**

To evaluate mRNA expression level of human osteocalcin, RNA was extracted by using TRIreagent (Invitrogen, ThermoFisher Scientific, Waltham, MA) . cDNA was synthesized from RNA by using a commercially available cDNA synthesis kit (Applied Biosystems, ThermoFisher Scientific, Waltham, MA) according to manufacturer’s instructions. Three steps cycling protocol (initial denaturation at 95°C for 10 min, 35 cycles of 15 s denaturation at 95°C, 30 s annealing at 60°C, and 30 s extension at 72°C) was used to amplify osteocalcin gene (forward primer: 5'-TGACGAGTTGGCTGACCA-3'; reverse primer: 5'-AGGGTGCCTGGAGAGGAG-3'). Relative fold difference between an experimental and calibrator sample was calculated by using comparative Ct (2-ΔΔCt) method [5]. 18S was used as internal standard to normalize the expression of the gene of interest.

**Flow cytometry of THP-1 cells.**

After the appropriate treatment cells were washed three times in PBS+1%FBS and then incubated for 45 minutes at 4°C in the dark with AF488-conjugated anti-human osteocalcin (R&D System, Minneapolis, MN, USA) and with APC-conjugated anti-human CD11b (BioLegend, San Diego, CA) according to manufacturer’s instructions. After incubation samples were washed other three times in PBS+1%FBS and then assessed by flow cytometry. Ten minutes before cell counts, cells were stained for viability with 7-aminoactinomycin D (7AAD). Doublets and nonviable cells were excluded as previously described. Osteocalcin positive cells were gated on the morphologic mononuclear cell fraction according to FMO controls.

1. Sun JK, Keenan HA, Cavallerano JD, et al (2011) Protection from retinopathy and other complications in patients with type 1 diabetes of extreme duration: the joslin 50-year medalist study. Diabetes Care 34:968–74. doi: 10.2337/dc10-1675

2. Feldman EL, Stevens MJ, Thomas PK, et al (1994) A practical two-step quantitative clinical and electrophysiological assessment for the diagnosis and staging of diabetic neuropathy. Diabetes Care 17:1281–9.

3. Levey AS, Stevens LA, Schmid CH, et al (2009) A new equation to estimate glomerular filtration rate. Ann Intern Med 150:604–12.

4. (1991) Fundus photographic risk factors for progression of diabetic retinopathy. ETDRS report number 12. Early Treatment Diabetic Retinopathy Study Research Group. Ophthalmology 98:823–33.

5. Schmittgen TD, Livak KJ (2008) Analyzing real-time PCR data by the comparative C(T) method. Nat Protoc 3:1101–8.
