# Supplementary material for: High density lipoprotein modulates osteocalcin expression in circulating monocytes: a potential protective mechanism for cardiovascular disease in type 1 diabetes
Source: Cardiovasc Diabetol. 2017 Sep 16;16:116. doi: 10.1186/s12933-017-0599-2 (PMC5602856; doi:10.1186/s12933-017-0599-2)
Supplement: Supplementary file 4 — Additional file 4: Table S1. Linear regression analysis for association between CD45_bright/CD14+/OCN+ (dependent variable) and different clinical and biochemical factors (independent variables). [file 12933_2017_599_MOESM4_ESM.docx]

| Independent variable | β | R^2^ | p-value |
| --- | --- | --- | --- |
| Gender (male vs female) | +0.052 | 0.104 | 0.082 |
| Age | +0.002 | 0.062 | 0.443 |
| Disease duration | +0.104 | 0.022 | 0.430 |
| BMI | +0.001 | 0.004 | 0.760 |
| Insulin dose | -0.127 | 0.007 | 0.288 |
| eGFR | -0.074 | 0.085 | 0.119 |
| HbA1c | -0.003 | 0.001 | 0.869 |
| Total cholesterol | -0.001 | 0.031 | 0.355 |
| Triglycerides | +0.001 | 0.026 | 0.393 |
| HDL | -0.002 | 0.180 | 0.019 |
| LDL | +0.001 | 0.016 | 0.510 |
| Trigl/HDL ratio | +0.047 | 0.099 | 0.090 |
| Corrected calcium | -0.082 | 0.085 | 0.119 |
| 25-OH Vitamin D | -0.027 | 0.015 | 0.519 |
| Alkaline phosphatase | -0.027 | 0.014 | 0.538 |
| hsCRP | -0.020 | 0.028 | 0.394 |
| Lipid lowering agents (Yes vs No) | +0.065 | 0.104 | 0.087 |
| Anti-hypertensive drugs (Yes vs No) | +0.046 | 0.067 | 0.175 |

**Table S1.**
